# Recognition of Dimethylarginine Analogues by Tandem Tudor Domain Protein Spindlin1

**DOI:** 10.3390/molecules27030983

**Published:** 2022-02-01

**Authors:** Miriam R. B. Porzberg, Laust Moesgaard, Catrine Johansson, Udo Oppermann, Jacob Kongsted, Jasmin Mecinović

**Affiliations:** 1 Department of Physics, Chemistry and Pharmacy, University of Southern Denmark, Campusvej 55, 5230 Odense, Denmark; porzberg@sdu.dk (M.R.B.P.); moesgaard@sdu.dk (L.M.); kongsted@sdu.dk (J.K.); 2 Botnar Research Centre, Nuffield Department of Orthopaedics, Rheumatology and Musculoskeletal Sciences, NIHR Bio-Medical Research Centre, University of Oxford, Oxford OX3 7LD, UK; catrine.johansson@ndorms.ox.ac.uk (C.J.); udo.oppermann@ndorms.ox.ac.uk (U.O.)

**Keywords:** epigenetics, histone, arginine methylation, molecular recognition, reader protein

## Abstract

Epigenetic readout of the combinatorial posttranslational modification comprised of trimethyllysine and asymmetric dimethylarginine (H3K4me3R8me2a) takes place via biomolecular recognition of tandem Tudor-domain-containing protein Spindlin1. Through comparative thermodynamic data and molecular dynamics simulations, we sought to explore the binding scope of asymmetric dimethylarginine mimics by Spindlin1. Herein, we provide evidence that the biomolecular recognition of H3K4me2R8me2a is not significantly affected when R8me2a is replaced by dimethylarginine analogues, implying that the binding of K4me3 provides the major binding contribution. High-energy water molecules inside both aromatic cages of the ligand binding sites contribute to the reader–histone association upon displacement by histone peptide, with the K4me3 hydration site being lower in free energy due to a flip of Trp151.

## 1. Introduction

**Figure 1:**
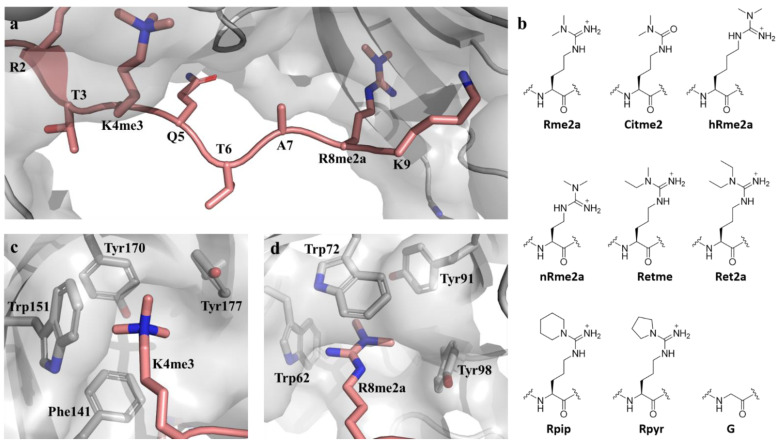
(**a**) Crystal structure of Spindlin1 containing the two neighbouring aromatic cages bound to H3K4me3R8me2a (PDB-ID: 4MZF); (**b**) Exploring the binding scope of Spindlin1 by incorporating asymmetric dimethylarginine analogues into the natural histone 3 sequence; (**c**) Phe141, Trp151, Tyr170, and Tyr177 form Tudor domain II binding cage that harbours K4me3; (**d**) R8me2a is encapsulated by Tudor domain I and consists of residues Trp62, Trp72, Tyr91, and Tyr98 (PDB-ID: 4MZF).

Posttranslational modifications on histones are key in regulating global chromatin environments in the human genome [1,2]. Among these modifications, the methylation of lysine and arginine residues in highly conserved histone tails has been extensively studied [3,4]. SAM-dependent methylation of the positively charged guanidinium group of arginine is catalysed by Protein Arginine Methyltransferases (PRMTs), of which nine are currently identified, and results in three methylation products: monomethylarginine (Rme1), symmetric dimethylarginine (Rme2s), and asymmetric dimethylarginine (Rme2a) [5]. Thereby, the positive charge and planar geometry of the guanidinium group are retained, and the potential hydrogen bond donors are decreased to four in Rme1 and three in Rme2s/a [4]. A histone arginine demethylase has not yet been identified; however, several non-heme 2-oxoglutarate- and Fe(II)-dependent JmjC Lysine Demethylases (KDMs) have shown arginine demethylation activity [6,7,8]. Methylation marks on histones serve as docking sites for reader proteins that bind these modifications with high affinity and specificity, thereby contributing to the regulation of the epigenetic landscape [9]. Dimethylarginine binding reader proteins contain Tudor domains possessing aromatic cages that associate with the positively charged guanidinium group through a combination of cation–π interactions and π–π stacking [10,11,12,13]. The distinction between the different methylation states is, among others, achieved by the size of the aromatic cage: methylarginine reader domain cages are usually narrower than methyllysine reader cages [10]. Furthermore, Rme2a is usually bound in a “cavity insertion” mode, while the larger and bulkier Rme2s is bound in a “surface groove” mode [14]. Methylarginine recognition has been studied by mutation of the aromatic residues in the aromatic cage, while binding scope and mechanism is poorly understood [12]. Among reader proteins with the highest binding affinities towards asymmetric dimethylarginine is Spindlin1, which is a 30 kDa homodimer composed of three Spin/Ssty domains [14]. Spindlin1 is associated with tumorigenesis and has been found to be highly expressed in various human cancer cells, including breast, ovarian, and colon cancer [15,16]. Furthermore, Spindlin1 stimulates rRNA expression and affects Wnt-signalling [14,17]. The overall structure of Spindlin1 is similar to other Tudor domain proteins, as the Spin/Ssty domains contain β-barrel folds that form two aromatic pockets enabling binding to a 10-mer H3K4me3R8me2a peptide with a K_d_ of 45 nM (Figure 1a) [14].

The so-called tandem Tudor domains vary slightly in their arrangement: while Tudor domain II (which binds K4me3) consists of Phe141, Trp151, Tyr170, and Tyr177, Tudor domain I is composed of Trp62, Trp72, Tyr91, and Tyr98, which are required for binding of R8me2 with a preference of asymmetric dimethylarginine (K_d_ = 45 nM) over symmetric dimethylarginine and monomethylarginine (K_d_ = 66 nM and K_d_ = 139 nM, respectively) (Figure 1c,d) [14,18]. Next to H3K4me3R8me2a, H4K20me3 has been identified as a binding site for Tudor domain II with a Kd of 0.8 μM. While H4K20me3R23me2a binds to Tudor domain I and II simultaneously, an eight-fold decrease in binding affinity indicates that H4K20me3 recognition by Tudor domain II is the primary binding site of Spindlin1 [19]. Furthermore, it was recently found that Spindlin1 is a reader of K4me3K9me3, with a binding affinity of K_d_ = 16 nM. Herein, K4me3 was identified as the primary binding site, while K9me3 recognition enhances the binding. Compared to R8me2a, K9me3 is better encapsulated in the aromatic pocket of Tudor domain I, indicating that methylarginine is only a minor binding mark of the methyl-lysine reader [20]. With Spindlin1 being associated with tumorigenesis as one of the most prominent histone readers of asymmetric dimethylarginine recognition, we sought to expand the binding scope by exploiting dimethylarginine analogues.

## 2. Results

For an improved understanding of the Spindlin1 binding mechanism, we evaluated several asymmetric dimethylarginine analogues by employing comparative isothermal titration calorimetry studies (Figure 1b). Herein, we designed and synthesised a panel of nine histone H3 peptides bearing trimethyllysine at position 4 and dimethylarginine analogues at position 8: Dimethylcitrulline (Citme2) is a novel Rme2a analogue, which lacks the positive charge at the guanidinium group that is proposed to enhance binding to Tudor domain I via cation–π interactions. Furthermore, we aimed to study the effect of side-chain length (hRme2a, nRme2a) and bulkiness of the methyl groups on binding by replacing one or both methyl groups with sterically more demanding ethyl group(s) (Retme, Ret2a) and by connecting the methyl group, resulting in bulky five- and six-membered ring structures (Rpip, Rpyr).

**Figure 2:**
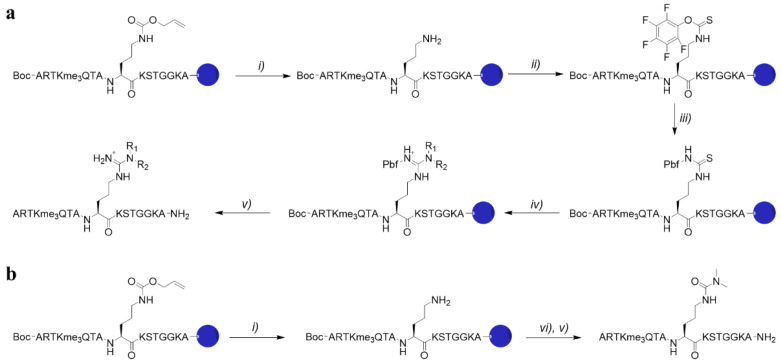
(**a**) Synthesis of H3K4me3R8me2a analogues on TentaGel resin: *i)* 24 eq. phenylsilane, 1 eq. tetrakis(triphenylphosphine)palladium(0), DCM, 1 h. *ii)* 5 eq. pentafluorophenyl chlorothionoformate, 10 eq. DIPEA, DCM, 1 h. *iii)* 5 eq. Pbf-NH_2_, 5 eq. potassium tert-butoxide, DMSO, 1 h. *iv)* 10 eq. amine, 7 eq. DIPEA, 5 eq. EDCI HCl. *v)* TFA/TIPS/H_2_O (95:2.5:2.5); (**b**) Synthesis of H3K4me3Cit8me2 on TentaGel resin: *vi)* 1.5 eq. dimethylcarbamoyl chloride, 1.5 eq. triethylamine, DMF, O/N.

To compare the binding affinity of Rme2a to its mimics, we chose the 15-mer histone 3 peptide (residues 1–15) as a ligand. As Spindlin1 binding towards H3R8me2a shows a ~500-fold enhanced binding affinity when the histone peptide contains the additional K4me3 residue, a panel of asymmetric dimethylarginine analogues was incorporated in 15-mer H3K4me3R8me2a peptides, thereby replacing the natural R8me2a and keeping K4me3 unmodified. Except for H3K4me3R8me2a and H3K4me3G8, histone peptides were synthesised by SPPS on TentaGel Resin using Fmoc-Orn(Alloc)-OH/Fmoc-Dab(Alloc)-OH/Fmoc-Lys(Alloc)-OH on position 8 for the subsequent on-resin synthesis of the unnatural dimethylarginine residues. The fully protected H3 peptide was selectively deprotected, yielding ornithine with a primary terminal amine group that was reacted with pentafluorophenyl chlorothionoformate and DIPEA in DCM for 1 h. Completion of the reaction was indicated by the Kaiser test, followed by a reaction with Pbf-NH_2_ and KO*t*Bu in DMSO for 1 h. Subsequently, the resin was functionalised with the desired secondary amine in the presence of EDCI and DIPEA in DMF overnight (Figure 2a). The histone 3 peptide bearing dimethylcitrulline was obtained after selective ornithine deprotection and on-resin reaction with dimethylcarbamoyl chloride and triethylamine in DMF overnight (Figure 2b). All histone peptides were cleaved off the resin using a mixture of TFA, TIPS, and water and further purified by RP-HPLC.

**Figure 3:**
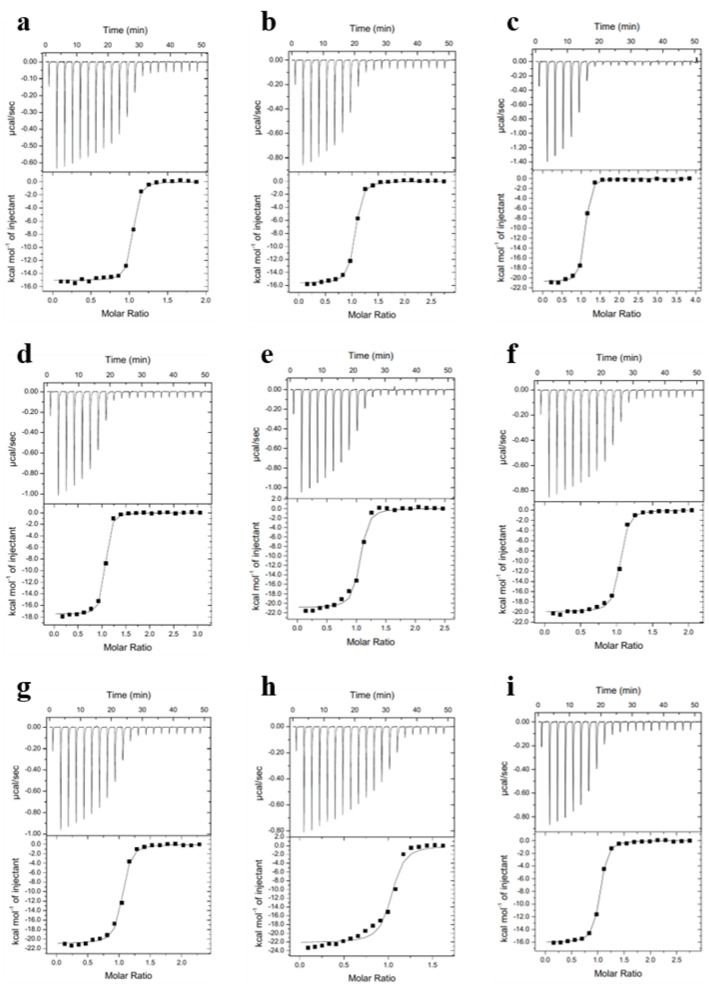
ITC curves showing (**a**) H3K4me3R8me2a; (**b**) H3K4me3Cit8me2; (**c**) H3K4me3hR8me2a; (**d**) H3K4me3nR8me2a; (**e**) H3K4me3R8etme; (**f**) H3K4me3R8et2a; (**g**) H3K4me3R8pip; (**h**) H3K4me3R8pyr; (**i**) H3K4me3G8 binding to Spindlin1.

Through isothermal titration calorimetry (ITC), we obtained thermodynamic parameters for the association of Spindlin1 with the H3K4me3R8me2a peptide and its analogues containing altered dimethylarginine residues. Spindlin1 was dialysed against ITC buffer containing 100 mM NaCl and 20 mM Tris HCl buffer (pH 8.0). Surprisingly, ITC binding curves were highly similar among all nine peptides, thereby revealing binding affinities in the nM range for all Rme2a analogues (Figure 3, Table 1).

ITC results revealed a K_d_ of 28 nM for the 15-mer H3K4me3R8me2a, which is in line with previous studies of the truncated 10mer peptide [15]. Additional thermodynamic parameters indicated that binding to Spindlin1 was enthalpy-driven (ΔH° = −17.3 kcal/mol), with the binding entropy being unfavourable (−TΔS° = 7.0 kcal/mol). This resulted in a free energy of ΔG° = −10.3 kcal/mol (Table 1). We hypothesised that cation–π interactions between the positively charged guanidinium group of the dimethylarginine and the aromatic binding cage contribute to a favourable binding. However, ITC results with H3K4me3Citme2, which is the most similar to H3K4me3R8me2a but lacks the positive charge, revealed similar thermodynamic binding parameters: while ΔG° is comparable to R8me2a, a ~2.9-fold decrease in binding affinity was observed (Kd = 81 nM). Furthermore, an enthalpy-entropy compensation was found, meaning that ΔH° becomes less favourable (ΔΔH° = 1.7 kcal/mol), which might have been due to the lack of favourable electrostatic interactions between the positively charged guanidinium group and the aromatic binding pocket, while −TΔS° became slightly more favourable (−TΔΔS° = −1.1 kcal/mol). ITC results of H3K4me3G8 with Spindlin1 revealed a Kd of 80 nM and very similar thermodynamic binding parameters, suggesting that the dimethylcitrulline side chain does not contribute significantly to the overall binding of the histone 3 peptide to Spindlin1. Furthermore, for all other dimethylarginine analogues, the thermodynamic binding parameters were in the same range as for H3K4me3R8me2a (Kd = 42–102 nM, ΔG° = −10.1–−9.5 kcal/mol, ΔH° = −22.5–−16.8 kcal/mol, −TΔS° = 5.9–13.0 kcal/mol), without any clear, visible trend. Notably, associations of all dimethylarginine analogues were more enthalpy-driven than both H3K4me3Citme2 and H3K4me3G8, while some were even more enthalpy-driven than observed in the binding of H3K4me3R8me2a to Spindlin1 (e.g., hR8me2a, R8etme, R8et2a, R8pip, R8pyr).

Taken together, these results indicate that the electrostatic interaction between the positively charged guanidinium group and the aromatic cage, as well as the presence of the hydrophobic arginine side chain, contribute to the favourable binding enthalpy we observed. However, these slight enthalpy changes coincided with entropy compensation, resulting in no significant improvements in binding affinity. This might have been due to K4me3 being the major binding mark, overshadowing the contribution of R8me2a to the overall binding of the 15-mer H3 peptide to Spindlin1.

**Figure 4:**
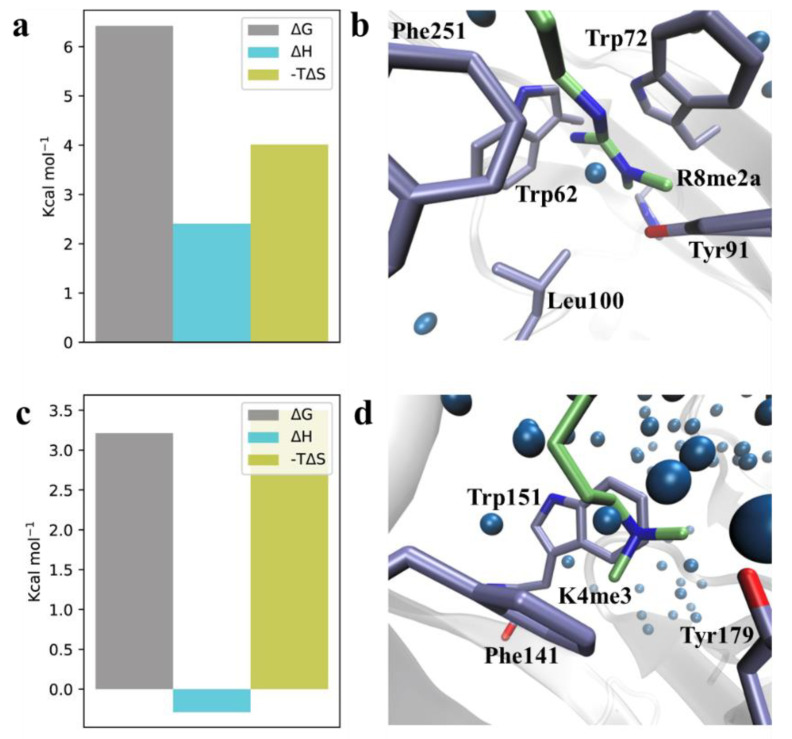
Hydration sites and estimated thermodynamic terms for the solvation of (**a**,**b**) Tudor domain I and (**c**,**d**) Tudor domain II aromatic cages of Spindlin1 (PDB-ID: 4MZF).

Next, we used molecular dynamic (MD) simulations and thermodynamic calculations to investigate the effect of aromatic cage desolvation for both Tudor domain I and II when bound to H3K4me3R8me2a. Calculations were performed using the grid inhomogeneous solvation theory (GIST) [21], which is a method for calculating entropic and enthalpic thermodynamic properties for grid-points inside the simulation box. The calculations allow for a detailed thermodynamic analysis of high-energy water molecules located on the inside of both aromatic cages and, thus, give an insight into the binding modes of K4me3/R8me2a. The contribution of aromatic cage desolvation of Tudor domain I resulted in an estimated change in total free energy of ΔG° = −6.4 kcal/mol upon binding, whilst a total desolvation free energy change of ΔG° = −3.2 kcal/mol was found in the case of Tudor domain II (Figure 4). These results indicate that both aromatic cages are occupied by high-energy water molecules. However, the R8me2a hydration site is twice as high in total free energy and, thus, more unfavourable.

Additional thermodynamic parameters of the hydration sites revealed that in both aromatic cages, solvation of the hydration site was entropically disfavoured to a similar degree, with −TΔS° = 4.0 kcal/mol and −TΔS° = 3.5 kcal/mol for R8me2a and K4me3, respectively. Surprisingly, the K4me3 aromatic cage revealed a slightly favourable enthalpy of the hydration site, while in the case of R8me2a, the hydration site was enthalpically unfavourable for water molecules, resulting in a stronger favourable free energy change from water displacement. This was most likely due to favourable interactions with the negatively charged Asp95. The observation that high-energy water molecules occupy the Kme3 aromatic cage is in agreement with recent studies on related reader proteins [22,23].

**Figure 5:**
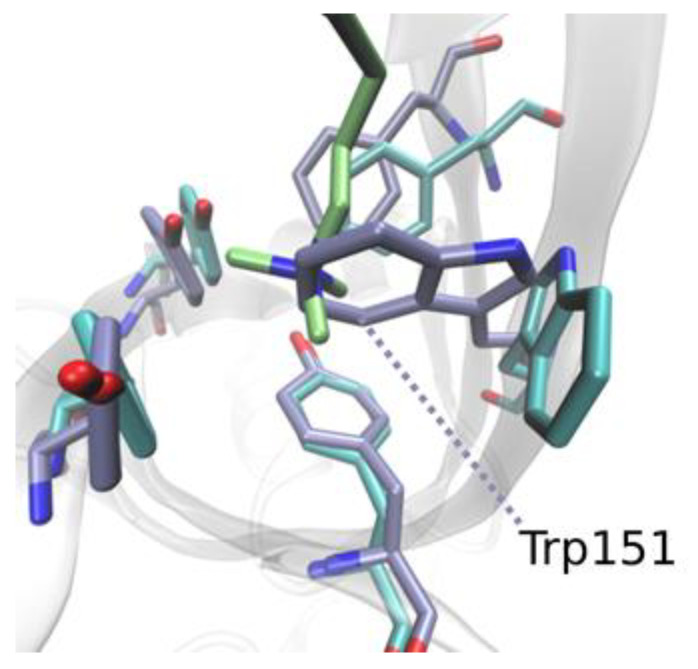
Molecular dynamics simulations reveal flip of Trp151 in the K4me3 aromatic cage.

To elucidate the differences in thermodynamic desolvation parameters, we investigated the nature of the aromatic binding sites. A comparison of Tudor domain II prior to binding and upon binding to K4me3 revealed a 72° flip of Trp151, while Phe141, Tyr170, and Tyr177 remained unaffected (Figure 5 and Figure S10). This flip was reflected by the increase in the RMSF around this residue (Supplementary Materials: Figure S11). When unoccupied, the volume of the aromatic K4me3 binding site decreased significantly through the flipped Trp151. Thus, the aromatic binding site was occupied by lower energy water molecules compared to the R8me2a binding site. Releasing water from Tudor domain I upon the binding of R8me2a made a more positive contribution to the biomolecular recognition event of Spindlin1 binding to histone 3. Overall, these results confirm the contribution of high-energy water molecules inside both aromatic pockets that possess unfavourable free energies. Displacing these water molecules through the binding of both K4me3 and especially R8me2a delivered a favourable contribution to the protein–ligand association.

## 3. Discussion

To elucidate dimethylarginine binding to the tandem Tudor-like reader protein Spindlin1, we herein describe synthesis and biomolecular recognition studies of novel dimethylarginine analogues that bind with nM binding affinities. While several arginine analogues, among which *N*^δ^-methylated arginine, *N*^ω^-alkylated arginines, *N*^ω^-hydroxy-arginine, and thiocitrulline have been evaluated as inhibitors and substrates for nitric oxide synthase, synthetic approaches towards the synthesis of dimethylarginine analogues are elaborate [24,25,26]. Recognition of dimethylarginine analogues incorporated into synthetic histone peptides has not been studied, with the exemption of alkylated Rme2a synthesised site-specifically from cysteine, which was applied to investigate recognition by TDRD3 [27]. The synthetic approach we present here is not limited to Spindlin1 but may also be a useful tool towards better understanding various other dimethylarginine reader proteins. The binding studies we present also revealed that the absence of the positively charged guanidinium group or the dimethylarginine side chain, as well as the replacement of the methyl groups by bulky moieties, resulted in a ~1.5–~3.7-fold decrease in the binding affinity of Spindlin1. Furthermore, we provide evidence that both aromatic binding sites are occupied by high-energy water molecules, which are entropically disfavoured, and in the case of the R8me2a cage, also are unfavourable in enthalpy. We conclude that upon displacement of such water molecules by K4me3 and R8me2a, more favourable energy of association is gained, overall contributing to the reader–histone association. Compared to R8me2a, the K4me3 hydration site was lower in total free energy, which is due to a unique flip of Trp151 into the K4me3 binding pocket, resulting in a decreased hydration surface when the protein is unbound. Our findings indicate that binding mode of H3K4me3R8me2a to Spindlin1 is not significantly affected by changes of R8me2a, pointing out that mainly K4me3 contributes to Spindlin1 binding. In addition to molecular studies demonstrating the several reader domains recognise Kme3 analogues [22,28,29,30,31,32,33], the work presented here shows that Spindlin1 has an ability to recognise Rme2a analogues. Results are important from a basic molecular perspective as well as from a biomedical perspective, as Spindlin1 has emerged as a promising target for epigenetic drug discovery [34,35,36,37,38].

## 4. Materials and Methods

### 4.1. Synthesis of H3K4me3Cit8me2

Fully protected H3K4me3Orn(Alloc)8 on TentaGel resin was swollen in DCM under nitrogen bubbling for 15 min at room temperature. Alloc deprotection was carried out using 24 eq. phenylsilane and 1 eq. tetrakis(triphenylphosphine)palladium(0) in DCM for 1 h under nitrogen bubbling. The resin was extensively washed with DCM (3x), DMF (3x) and 5% sodium diethyldithiocarbamate in DMF, and completion of the reaction was monitored by Kaiser test. Subsequently, the resin was reacted with 1.5 eq. dimethyl carbamoyl chloride and 1.5 eq. triethylamine in DMF overnight. A negative Kaiser test indicated completion of the reaction, after which the resin was extensively washed with DMF (3x), DCM (3x), MeOH (3x) and Et_2_O, and dried in vacuo. The obtained peptide was cleaved off the resin and deprotected using TFA/TIPS/water (95:2.5:2.5) for 4 h at room temperature, followed by precipitation in ice-cold diethyl ether, freeze-drying, and purification by prep-HPLC (Figure S2).

### 4.2. Synthesis of H3K4me3X8

Fully protected histone peptide on TentaGel resin was swollen in DCM under nitrogen bubbling for 15 min at room temperature. Alloc deprotection was carried out using 24 eq. phenylsilane and 1 eq. tetrakis(triphenylphosphine)palladium(0) in DCM for 1 h under nitrogen bubbling. The resin was extensively washed with DCM (3x), DMF (3x), and 5% sodium diethyldithiocarbamate in DMF, and completion of the reaction was monitored by the Kaiser test. Subsequently, the resin was reacted with 5 eq. Pentafluorophenyl chlorothionoformate and 10 eq. DIPEA in DCM for 1h. A negative Kaiser test indicated completion of the reaction, after which the resin was extensively washed with DCM (3x) and DMSO (3x). The resin was then reacted with 5 eq. Pbf-NH_2_ and 5 eq. KO*t*Bu in DMSO for 1 h, followed by washing with DMSO (3x) and DMF (3x). Afterwards, the resin was reacted with 10 eq. secondary amine, 7 eq. DIPEA, and 5 eq. EDC HCl in DMF overnight. The resin was then extensively washed with DMF (3x), DCM (3x), MeOH (3x), and Et_2_O and dried in vacuo. The obtained peptide was cleaved, purified, and characterised as mentioned above (Figures S1,S3–S9).

### 4.3. Expression and Purification of Human Spindlin1

SPIN1_49-262_ was expressed and purified as described previously [35]. Briefly, His-tagged SPIN1 was expressed in E.coli strain BL21 at 37 °C until OD_600_ = 2.0. Protein expression was induced by the addition of 0.1 mM IPTG, followed by incubation at 18 °C overnight. Bacteria were harvested by centrifugation and subsequent resuspension of the pellet in buffer containing 50 mM Hepes pH 7.5, 500 mM NaCl, 10 mM Imidazole, 5% glycerol, 0.5 mM TCEP, and a protease inhibitor cocktail (Sigma, St. Louis, MO, USA). After lysis and centrifugation, His-tagged SPIN1 was affinity purified by Ni^2+^-NTA-agarose chromatography (Cytiva, Marlborough, MA, USA) applying a gradient of imidazole. Subsequent size exclusion chromatography (Superdex 75, Cytiva) was performed with buffer containing 10 mM Hepes pH 7.5, 500 mM NaCl, 5% glycerol, and 0.5 mM TCEP.

### 4.4. Isothermal Titration Calorimetry

Isothermal titration calorimetry was performed at 25 °C on a fully automated Microcal Auto-iTC200 (GE Healthcare Life Sciences, Marlborough, MA, USA). SPIN1_49-262_ was dialysed against ITC buffer containing 20 mM Tris, 100 mM NaCl pH 8.0. Titrations were carried out with an initial injection of 0.5 μL, followed by 19 injections of 2 μL at a total concentration of 20 μM SPIN1_49-262_ and 150-370 μM peptide. Fitting and analyses of the acquired titration curves were performed with Origin 6.0 (Microcal Inc., Northampton, MA, USA) using the One Set of Binding Sites model.

### 4.5. Molecular Dynamics Simulations

The crystal structure of Spindlin1 bound to histone H3(K4me3-R8me2a) was acquired from the Protein Data Bank (PDB ID: 4MZF, X-ray crystal structure resolution 2.10 Å) and imported into the Maestro module available with the Schrödinger suite [39]. Here, water, chloride, and histone peptide atoms were removed, and missing loops and side-chain atoms were added using the Prime tool available in the Protein Preparation Wizard of Maestro [40]. Protonation states at pH = 7.0 were determined using the PROPKA tool, which is also part of the Protein Preparation Wizard [41,42]. The MD simulation was performed using the Amber 18 software [43]. The tleap tool in Amber was used to solvate the system in water with a buffering distance of 12.0 Å to the protein and to add salt ions to neutralise the system and reach a concentration of 0.150 M. tleap was then used to create topology and coordinate files using the Amber ff14SB (protein and peptide) [44] and the TIP3P water model [45]. Energy minimizations were performed with constraints on heavy atoms and a maximum of 1000 cycles. The first 500 iterations were performed using the steepest descent algorithm, and the rest used the conjugate gradient algorithm. The system was then heated to 300 K in the span of 50 ps using the Langevin thermostat, followed by 50 ps of density equilibration using the Berendsen barostat [46,47]. The systems were then set to equilibrate (with production settings) for 500 ps at constant pressure and a temperature of 300 K. Initial velocities were generated from random seeds based on a Maxwell-Boltzmann distribution. Bonds involving hydrogen were constrained using the SHAKE algorithm [48]. All MD simulations were run using a timestep of 2 fs. Following this initial short equilibration step, a longer simulation of 100 ns was carried out with production settings (Figure S12). Finally, a simulation of 50 ns with all protein-heavy atoms restrained by a harmonic potential with a force constant of 100 kcal/mol/Å2 was performed to sample water dynamics. Coordinates were printed to the trajectory file every ps, which left 50,000 snapshots for water dynamic analysis. To analyse solvent accessible surface area (SASA) and thermodynamic properties of water, we used the gist (Grid Inhomogeneous Solvation Theory) Amber module with a grid spacing of 0.5 Å [21]. The predicted effect of having residues bind at the water cages was calculated by summing the thermodynamic potentials of the grid points of the simulation within the vdW area of non-backbone atoms of the histone in the aligned structure. Hydration sites were identified using the Placevent tool [49].

## 5. Conclusions

Through the synthesis of dimethylarginine analogues, comparative titration calorimetry, and molecular dynamics simulations, we conclude that the epigenetic reader protein Spindlin1 recognises several dimethylarginine analogues incorporated into the H3K4me3R8me2a peptide, revealing that K4me3 is the major contributor to Spindlin1 recognition. Furthermore, the displacement of high-energy water molecules upon binding of K4me3 and R8me2a to both aromatic cages results in favourable association energy.

## Figures and Tables

**Table 1:** Thermodynamic parameters for the binding of the 15-mer H3K4me3X8 peptides to Spindlin1. Data are mean ± SD, n = 0.98–1.08, T = 25.0 °C, values obtained from 3 repeated ITC experiments.

	K_d_ (nM)	ΔG°	ΔH°	−TΔS°
H3K4me3R8me2a	28 ± 0.5	−10.3 ± 0.02	−17.3 ± 1.9	7.0 ± 1.9
H3K4me3Cit8me2	81 ± 6	−9.7 ± 0.04	−15.6 ± 0.3	5.9 ± 0.2
H3K4me3hR8me2a	56 ± 15	−9.9 ± 0.2	−20.9 ± 0.7	11.0 ± 0.7
H3K4me3nR8me2a	42 ± 8	−10.1 ± 0.1	−16.9 ± 1.3	6.8 ± 1.2
H3K4me3R8etme	88 ± 6	−9.6 ± 0.1	−20.8 ± 0.6	11.2 ± 0.6
H3K4me3R8et2a	46 ± 4	−10.0 ± 0.1	−19.3 ± 0.6	9.3 ± 0.6
H3K4me3R8pip	81 ± 19	−9.7 ± 0.2	−20.9 ± 0.3	11.2 ± 0.1
H3K4me3R8pyr	102 ± 12	−9.5 ± 0.1	−22.5 ± 0.4	13.0 ± 0.5
H3K4me3G8	80 ± 15	−9.7 ± 0.1	−15.5 ± 0.5	5.8 ± 0.6

## Data Availability

The data presented in this study are available in Supplementary material.

## Supplementary Materials

Figure S1: (a) Analytical HPLC and (b) MALDI-TOF MS spectrum of H3K4me3R8me2a after RP-HPLC purification, Figure S2: (a) Analytical HPLC and (b) MALDI-TOF MS spectrum of H3K4me3Cit8me2 after RP-HPLC purification, Figure S3: (a) Analytical HPLC and (b) MALDI-TOF MS spectrum of H3K4me3hR8me2a after RP-HPLC purification, Figure S4: (a) Analytical HPLC and (b) MALDI-TOF MS spectrum of H3K4me3nR8me2a after RP-HPLC purification, Figure S5: (a) Analytical HPLC and (b) MALDI-TOF MS spectrum of H3K4me3R8etme after RP-HPLC purification, Figure S6: (a) Analytical HPLC and (b) MALDI-TOF MS spectrum of H3K4me3R8et2a after RP-HPLC purification, Figure S7: (a) Analytical HPLC and (b) MALDI-TOF MS spectrum of H3K4me3R8pip after RP-HPLC purification, Figure S8: (a) Analytical HPLC and (b) MALDI-TOF MS spectrum of H3K4me3R8pyr after RP-HPLC purification, Figure S9: (a) Analytical HPLC and (b) MALDI-TOF MS spectrum of H3K4me3G8 after RP-HPLC purification, Figure S10: Plot of the side-chain dihedral angle (θ) of Trp151 during the simulation. The dihedral angle is 73.9° in the crystal structure, Figure S11: RMSF plot of the backbone fluctuations during the unrestrained part of the unbound protein simulation, Figure S12: RMSD plot of the backbone movement during the unrestrained part of the unbound protein simulation.

## Sample Availability

Not available.
