# Supplementary material for: Recognition of Dimethylarginine Analogues by Tandem Tudor Domain Protein Spindlin1
Source: Molecules. 2022 Feb 1;27(3):983. doi: 10.3390/molecules27030983 (PMC8838590; doi:10.3390/molecules27030983)
Supplement: Supplementary file 1 [file molecules-27-00983-s001.zip › molecules-1551305-supplementary.pdf]

Supporting Information

Recognition of Dimethylarginine Analogues by Tandem Tudor Domain Protein Spindlin1

Miriam R.B. Porzberg [1], Laust Moesgaard [1], Catrine Johansson [2], Udo Oppermann [2], Jacob Kongsted [1] and Jasmin Mecinović [1,*]

[1] Department of Physics, Chemistry and Pharmacy, University of Southern Denmark, Campusvej 55, 5230 Odense M, Denmark

[2] Botnar Research Centre, Nuffield Department of Orthopaedics, Rheumatology and Musculoskeletal Sciences, NIHR Biomedical Research Centre, University of Oxford, OX3 7LD Oxford, UK

* Correspondence: mecinovic@sdu.dk; Tel.: +45-6550-3603

Table of Contents

1. General experimental

2. Synthesis of histone peptides

3. Characterisation of histone peptides

4. Supporting figures

1. General experimental

All chemicals are commercially available and were used without further purification. Fmoc-protected amino acids were purchased from Novabiochem (Merck-Millipore, Darmstadt, Germany), Fmoc-Lys(Me)3-OH hydrochloride was purchased from Fluorochem (Derbyshire, UK), Fmoc-ADMA(Pbf)-OH was purchased from Chem-Impex (Wood Dale, IL, USA), TentaGel HL RAM Resin (0.40 mmol/g) was purchased from Rapp Polymere (Tübingen, Germany). Peptides were purified via preparative reverse-phase HPLC (RP-HPLC) using H_2O with 0.1% TFA (buffer A) and ACN with 0.1% TFA (buffer B) on a Gemini 10 um NX-C18 110Å LC column (Phenomenex, Torrance, CA, USA). A VaCo2 lypholiser (Zirbus Technolgy GmbH, Bad Grund, Germany) was used for lyophilisation of the purified peptides. A Bruker autoflex II smartbeam and a Bruker microTOF Q II MALDI-TOF system with a matrix containing α-Cyano-4-hydroxycinnamic acid were used for mass spectrometry. The purified peptides were analysed with analytical RP-HPLC on a Gemini 5um C18 110Å LC column (Phenomenex) monitoring injections at 215 nm (1ml/min). MALDI-TOF mass spectra were acquired on a UltrafleXtreme-II tandem system (Bruker, Billerica, MA, USA), using α-Cyano-4-hydroxycinnamic acid matrix 1:1 (MQ and ACN, 0.1% TFA).

2. Synthesis of histone peptides

Histone 3 peptides for subsequent on-resin modification ($H3_{1-15}$Orn8) were prepared on TentaGel HL RAM resin (0.4 mmol/g loading) using microwave assisted SPPS on a Liberty Blue automated peptide synthesizer (CEM corporation, Matthews, NC, USA). H3K4me3G8 and H3K4me3R8me2a were synthesized partially via automated SPPS on Liberty Blue ($H3_{5-15}$ and $H3_{9-15}$ respectively), followed by manual SPPS using 3.0 eq of amino acids, 3.6 eq HOBt, 3.3 eq DIC in DMF for couplings for 2h at room temperature, and using 20% piperidine in DMF for deprotections for 30 minutes at room temperature. Amino acid couplings and Fmoc deprotections were followed by Kaiser test. Final peptides were cleaved off the resin and deprotected using TFA/TIPS/water (95:2.5:2.5) for 4h at room temperature, followed by precipitation in ice-cold diethyl ether.

3. Characterisation of histone peptides

	**Sequence**	**Formula**	***m/z* calc.**	**$[M+2H]^{2+}$ found**	**$[M+3H]^{3+}$ found**
H3K4me3R8me2a	ARTKme3QTAR^8me2aKSTGGKA	$C_{68}H_{128}N_{25}O_{21}^+$	1630.97	815.51	544.00
H3K4me3Cit8me2	ARTKme3QTACit^{8}me2KSTGGKA	$C_{68}H_{127}N_{24}O_{22}^+$	1631.96	815.98	544.32
H3K4me3hR8me2a	ARTKme3QTAhR^8me2aKSTGGKA	$C_{69}H_{130}N_{25}O_{21}^+$	1644.99	822.49	548.67
H3K4me3nR8me2a	ARTKme3QTAnR^8me2aKSTGGKA	$C_{67}H_{126}N_{25}O_{21}^+$	1616.96	808.48	539.32
H3K4me3R8etme	ARTKme3QTAR^8etmeKSTGGKA	$C_{69}H_{130}N_{25}O_{21}^+$	1644.99	822.49	548.66
H3K4me3R8et2a	ARTKme3QTAR^8et2aKSTGGKA	$C_{70}H_{132}N_{25}O_{21}^+$	1659.00	829.50	553.34
H3K4me3R8pip	ARTKme3QTAR^8pipKSTGGKA	$C_{71}H_{132}N_{25}O_{21}^+$	1671.00	835.50	557.34
H3K4me3R8pyr	ARTKme3QTAR^8pyrKSTGGKA	$C_{70}H_{130}N_{25}O_{21}^+$	1656.99	828.49	552.67
H3K4me3G8	ARTKme3QTAG^8KSTGGKA	$C_{62}H_{115}N_{22}O_{21}^+$	1503.86	751.93	501.62

a)

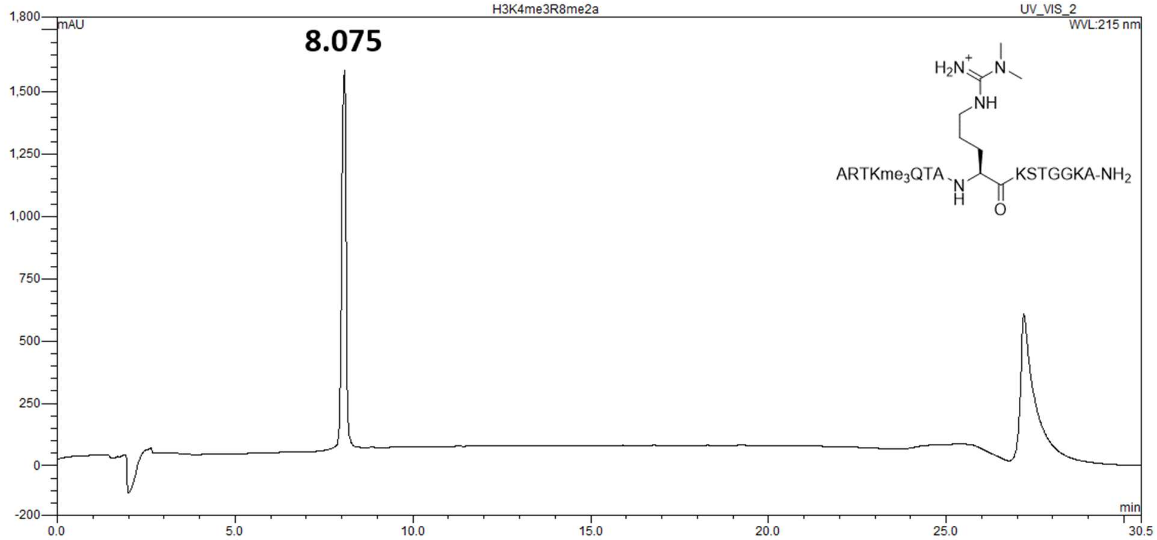

b)

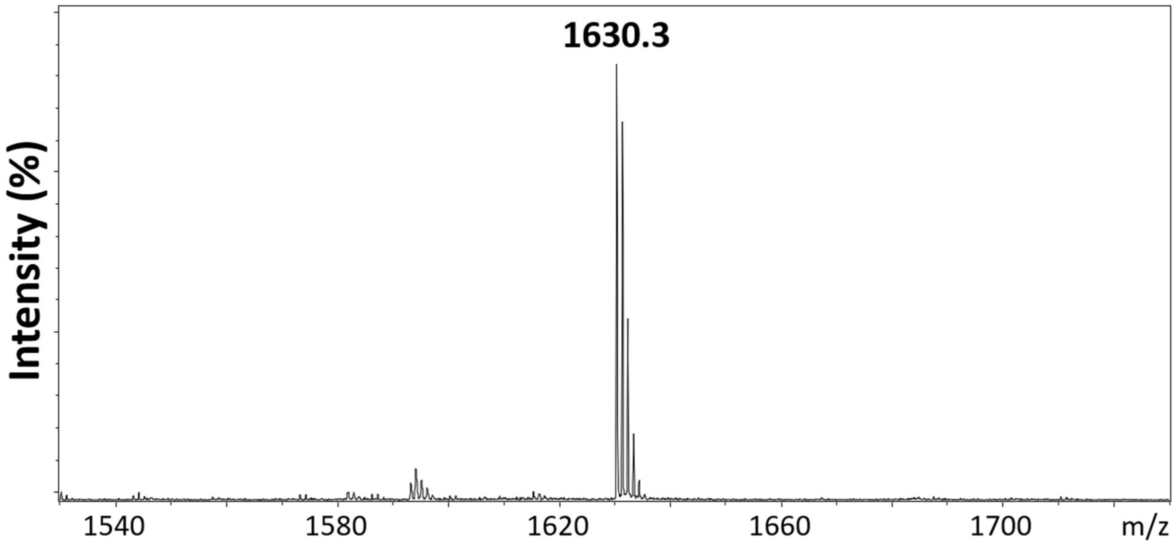

Figure S1. (a) Analytical HPLC and **(b)** MALDI-TOF MS spectrum of H3K4me3R8me2a after RP-HPLC purification.

a)

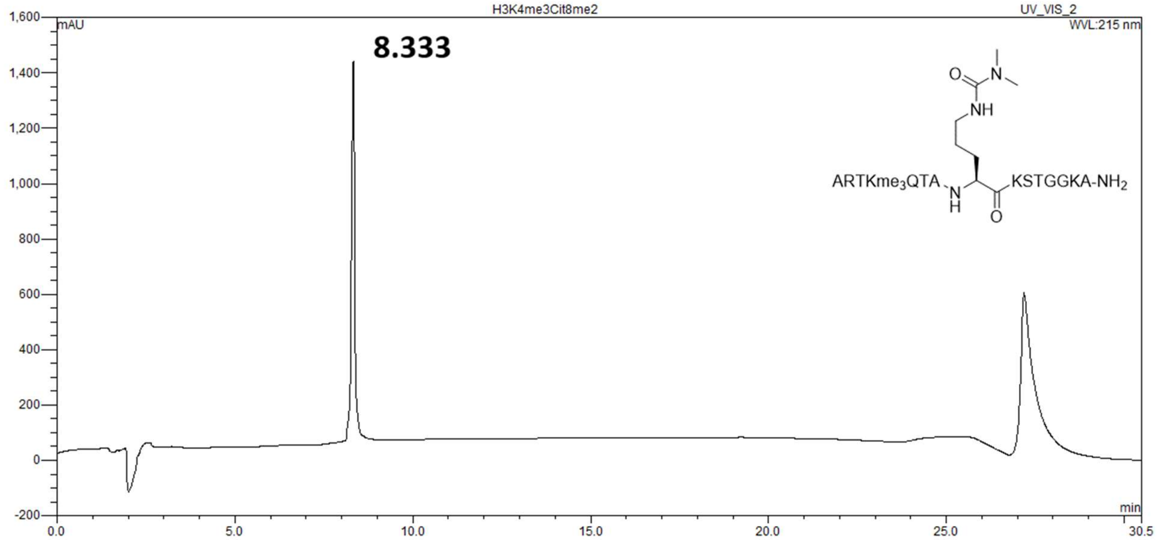

b)

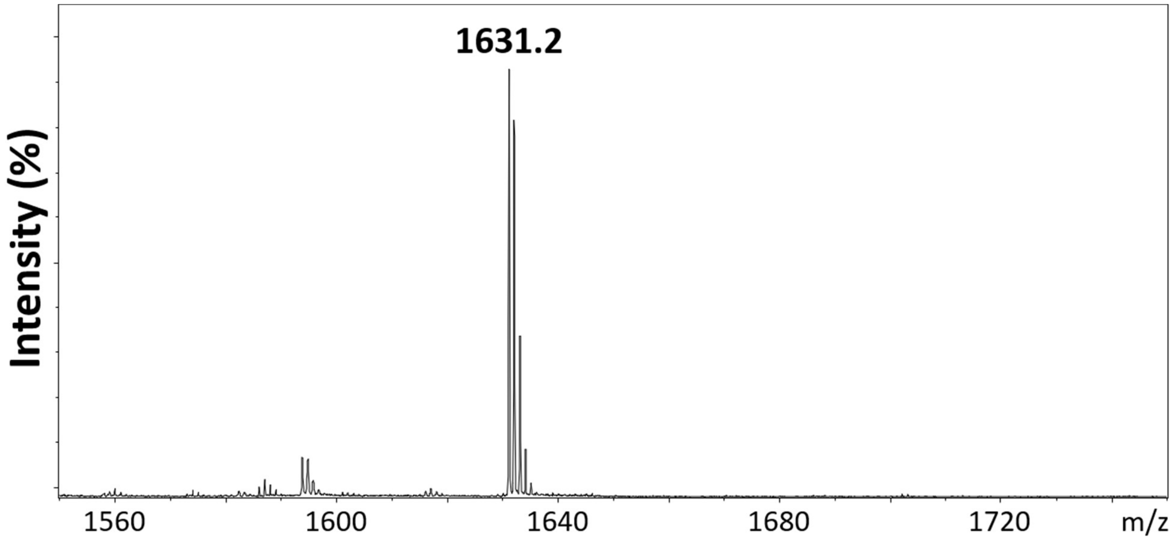

Figure S2. (a) Analytical HPLC and **(b)** MALDI-TOF MS spectrum of H3K4me3Cit8me2 after RP-HPLC purification.

a)

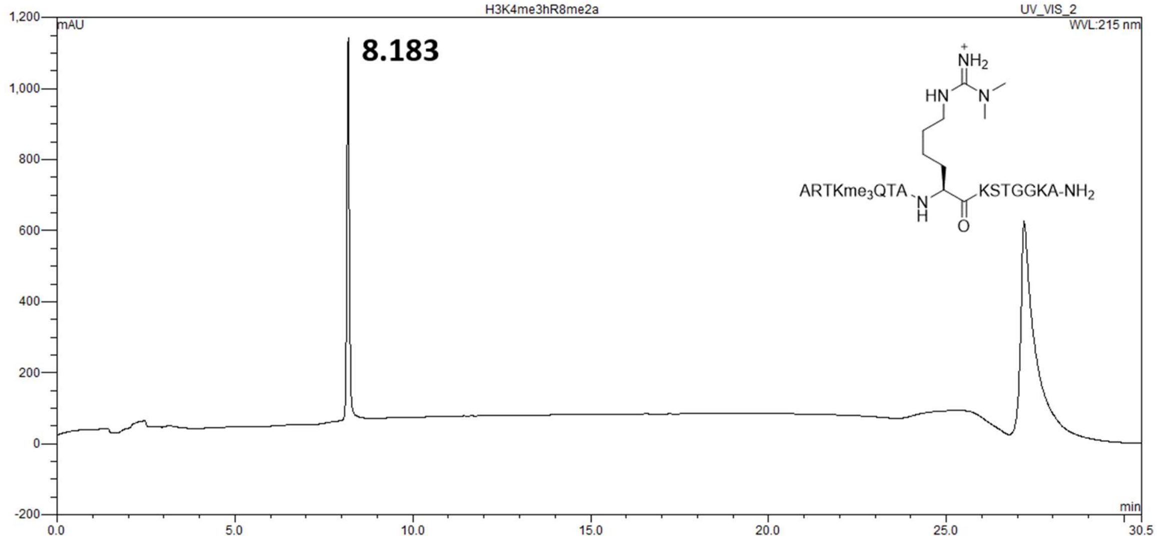

b)

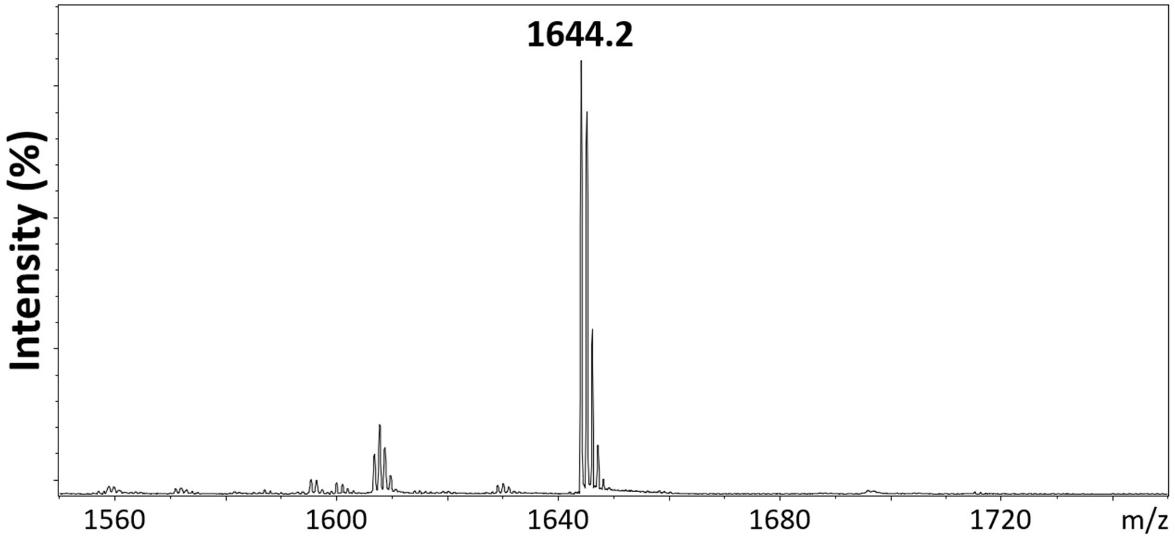

Figure S3. (a) Analytical HPLC and **(b)** MALDI-TOF MS spectrum of H3K4me3hR8me2a after RP-HPLC purification.

a)

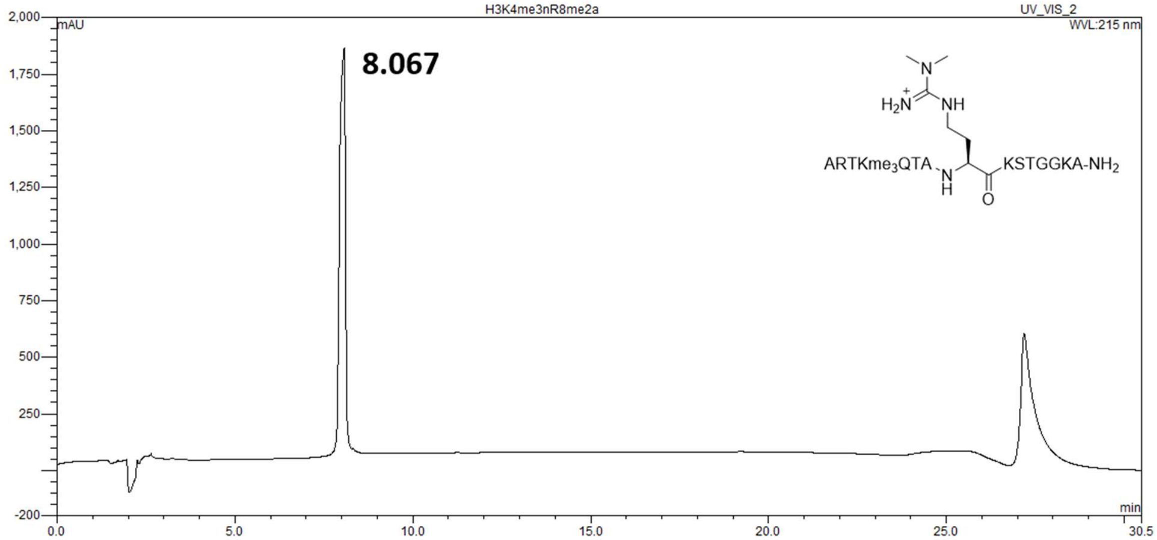

b)

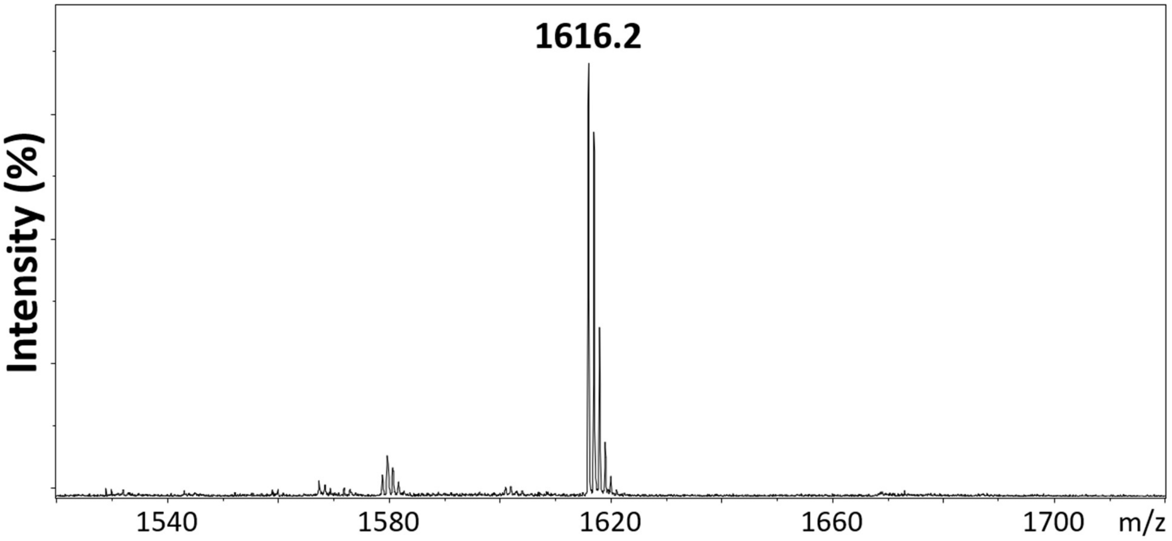

Figure S4. (a) Analytical HPLC and **(b)** MALDI-TOF MS spectrum of H3K4me3nR8me2a after RP-HPLC purification.

a)

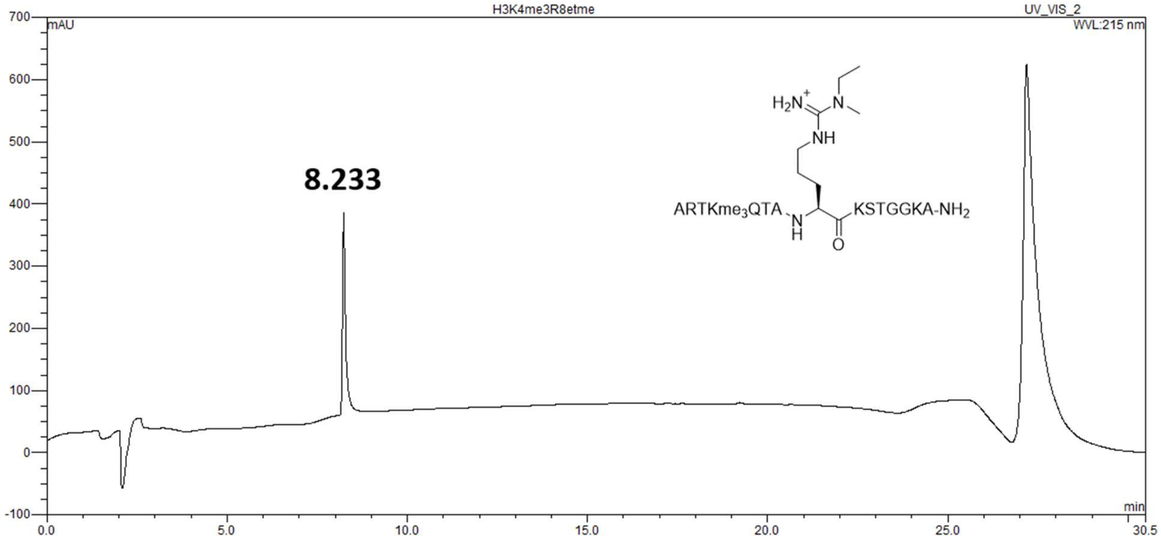

b)

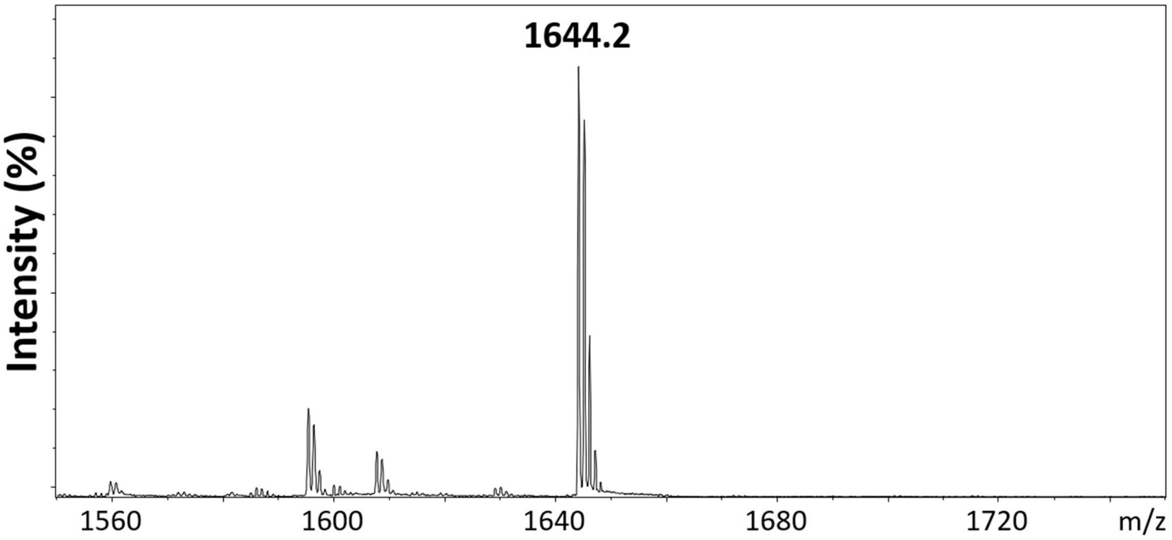

Figure S5. (a) Analytical HPLC and **(b)** MALDI-TOF MS spectrum of H3K4me3R8etme after RP-HPLC purification.

a)

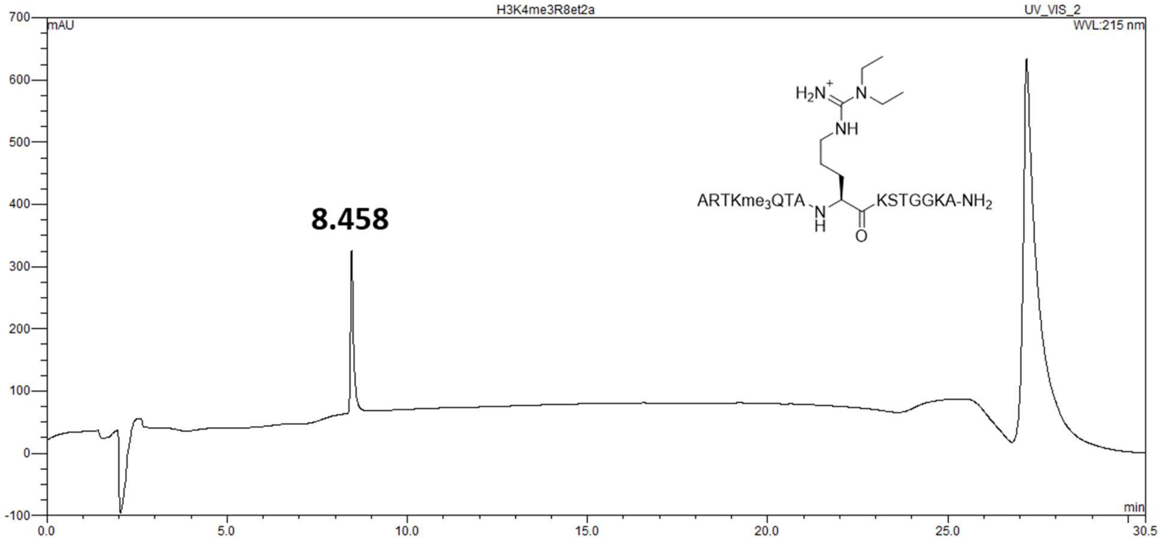

b)

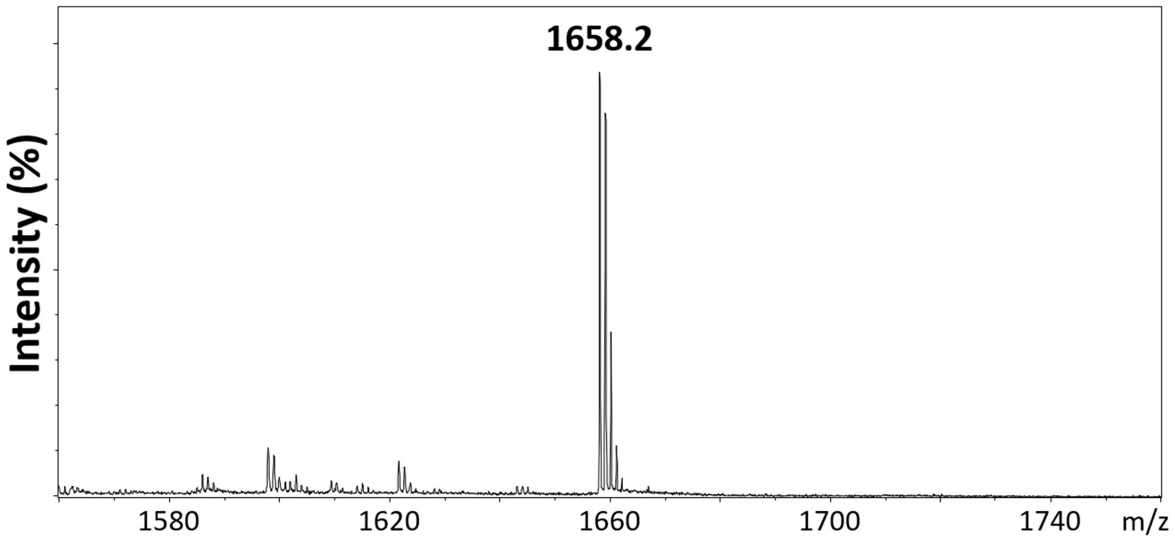

Figure S6. (a) Analytical HPLC and **(b)** MALDI-TOF MS spectrum of H3K4me3R8et2a after RP-HPLC purification.

a)

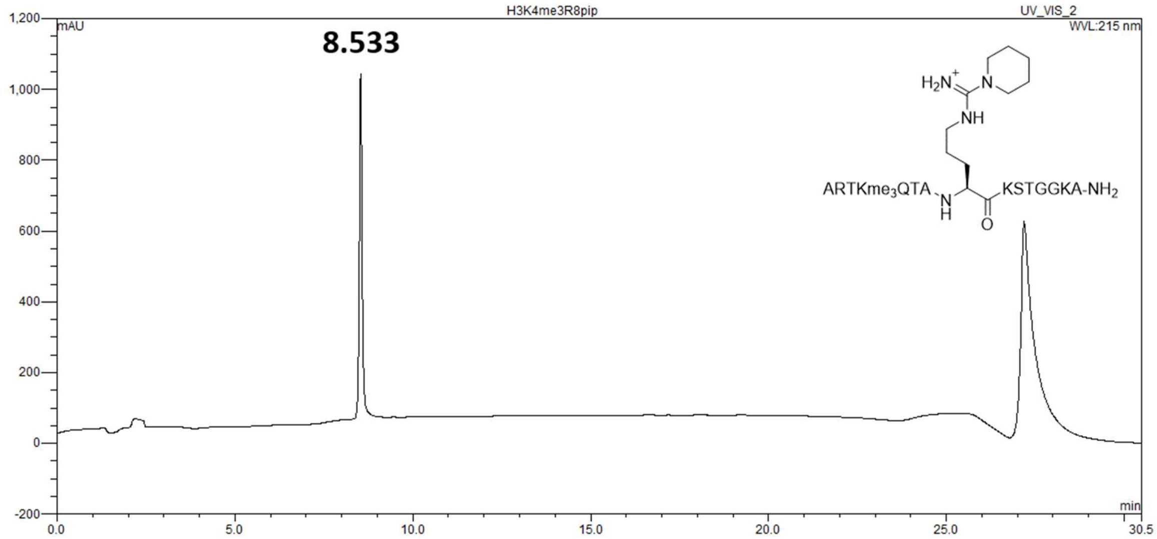

b)

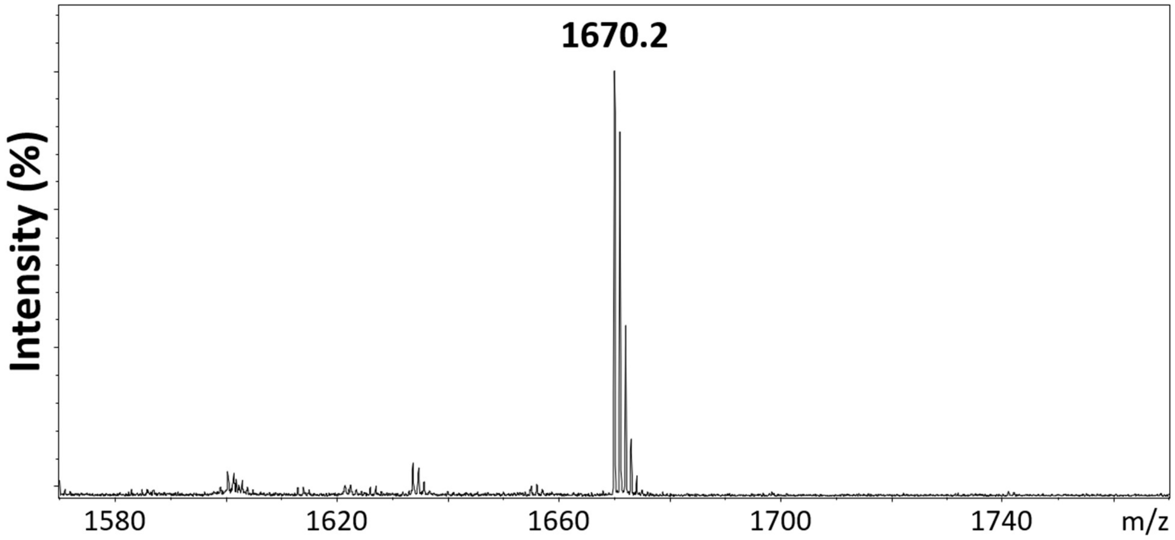

Figure S7. (a) Analytical HPLC and **(b)** MALDI-TOF MS spectrum of H3K4me3R8pip after RP-HPLC purification.

a)

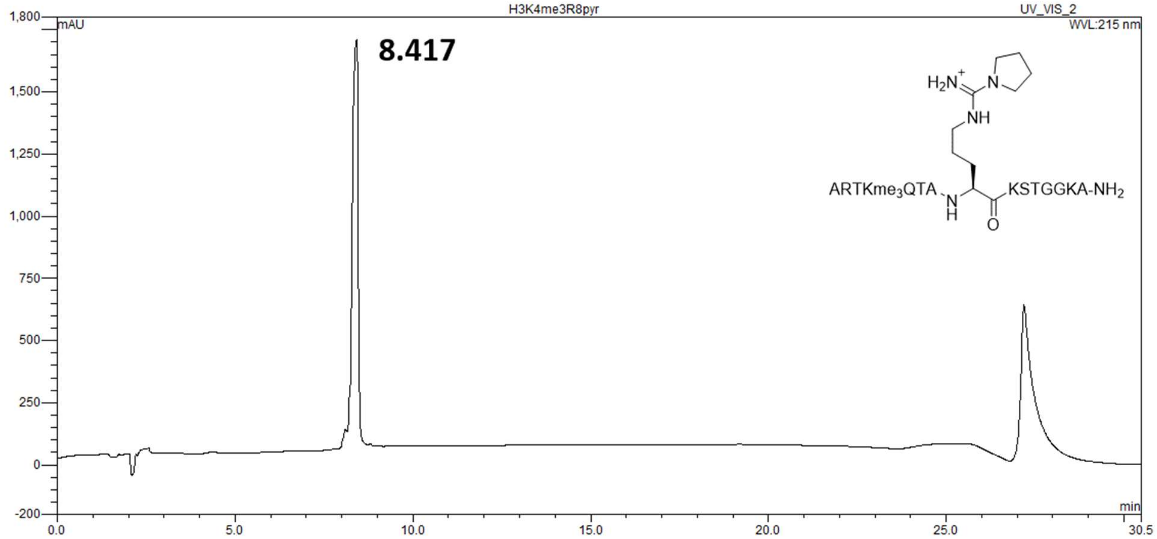

b)

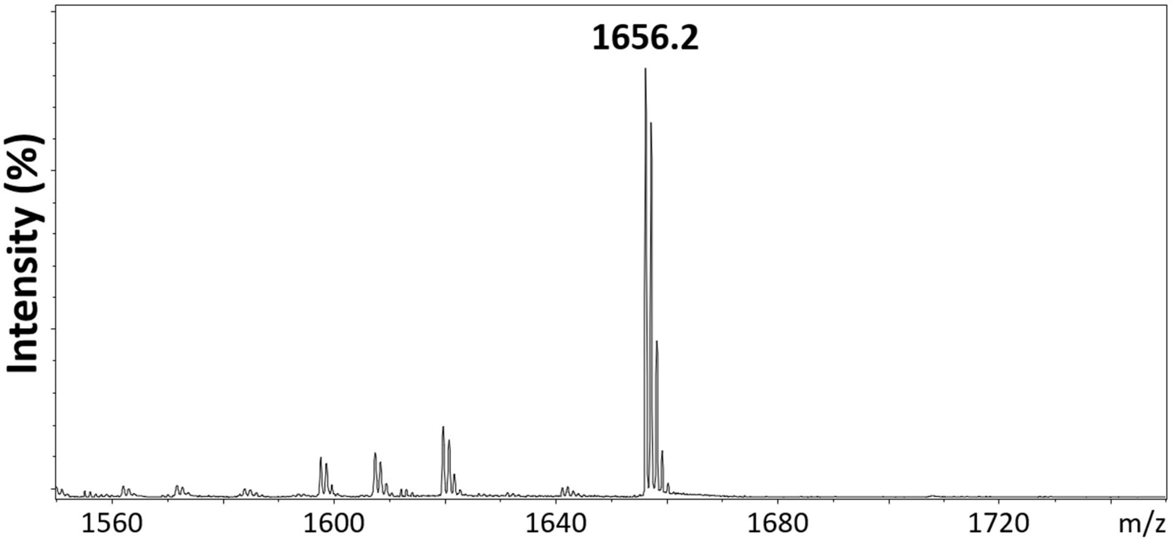

Figure S8. (a) Analytical HPLC and **(b)** MALDI-TOF MS spectrum of H3K4me3R8pyr after RP-HPLC purification.

a)

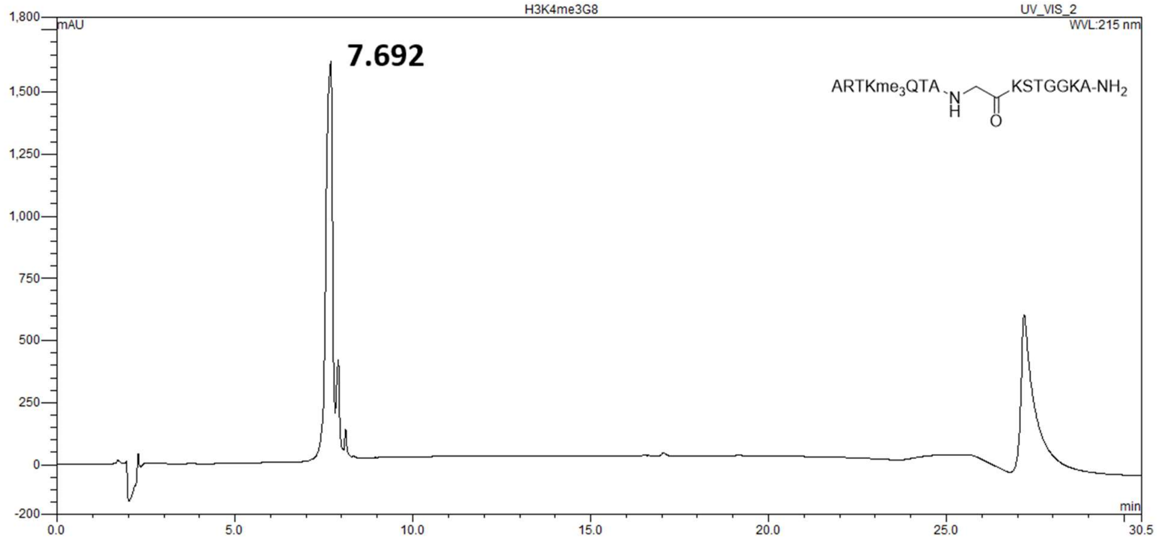

b)

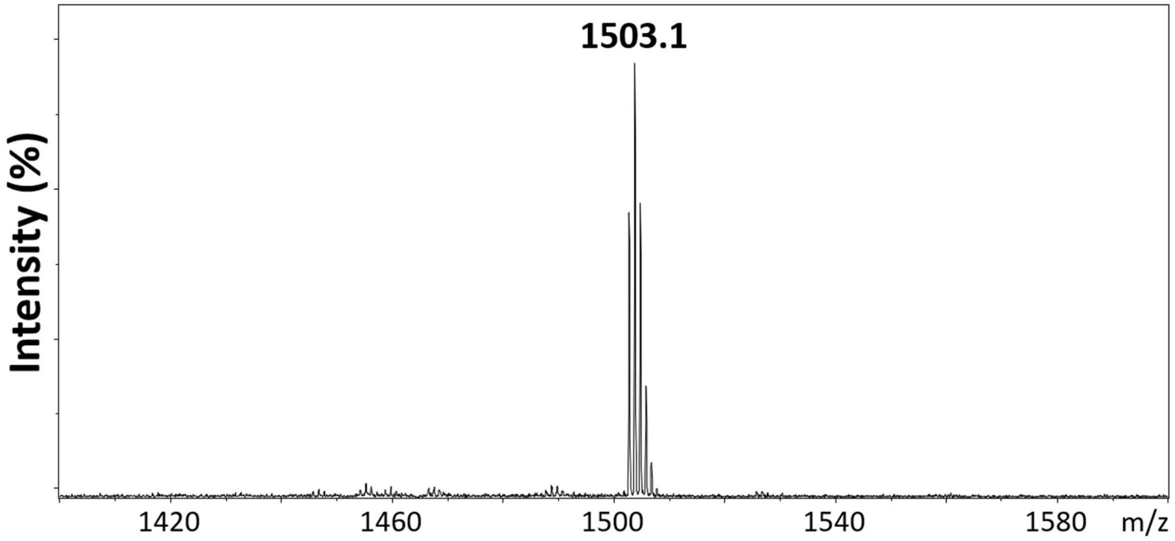

Figure S9. (a) Analytical HPLC and **(b)** MALDI-TOF MS spectrum of H3K4me3G8 after RP-HPLC purification.

4. Supporting figures

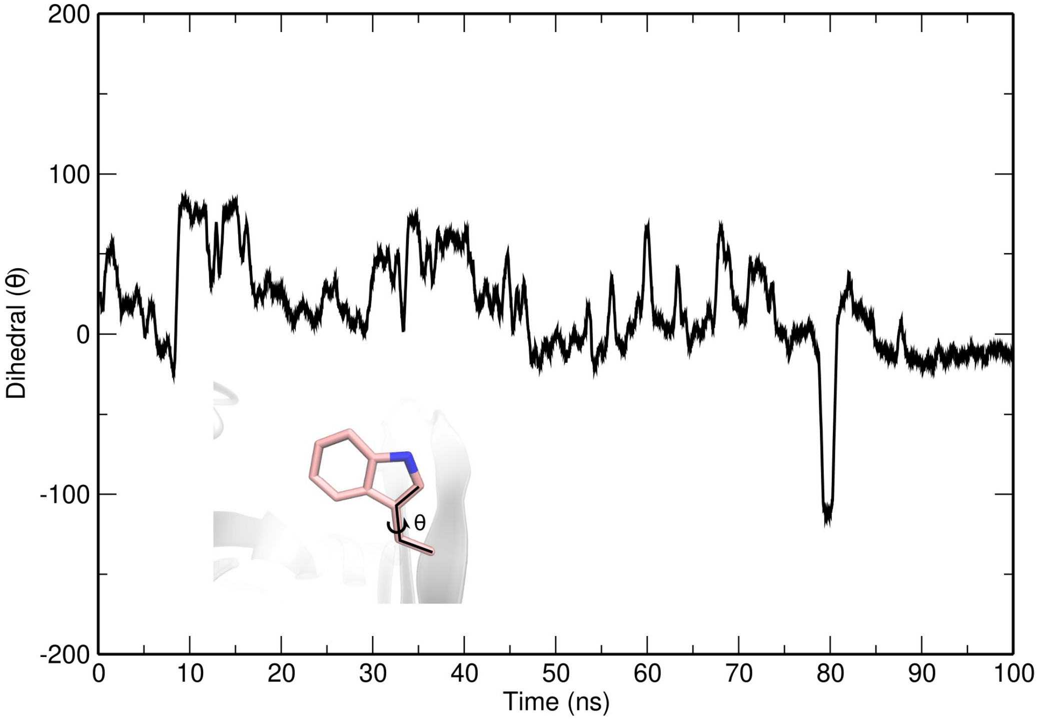

Figure S10. Plot of the side-chain dihedral angle (θ) of Trp151 during the simulation. The dihedral angle is 73.9° in the crystal structure.

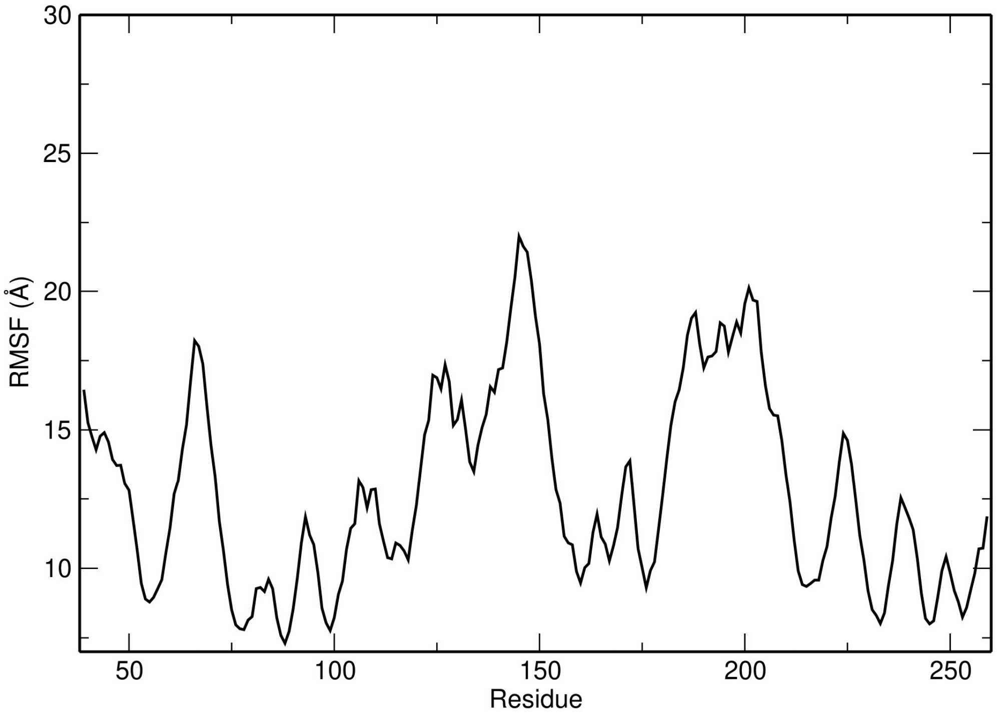

Figure S11. RMSF plot of the backbone fluctuations during the unrestrained part of the unbound protein simulation.

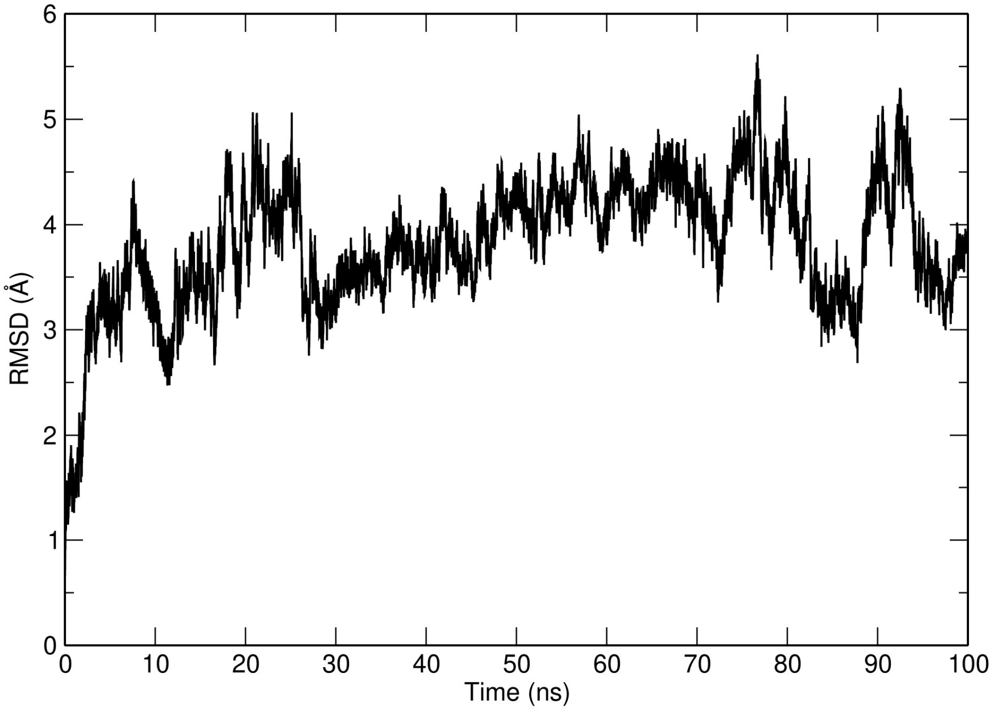

Figure S12. RMSD plot of the backbone movement during the unrestrained part of the unbound protein simulation.
